# Exploring the hidden treasure in arid regions: pseudocereals as sustainable, climate-resilient crops for food security

**DOI:** 10.3389/fpls.2025.1662267

**Published:** 2025-09-19

**Authors:** Ramya Manoharan, Sugandha Asthana, Chythra Somanathan Nair, Trupti Gokhale, Drishya Nishanth, Abdul Jaleel, Neeru Sood

**Affiliations:** 1 Department of Integrative Agriculture, College of Agriculture and Veterinary Medicine, United Arab Emirates University, Al Ain, United Arab Emirates; 2 Department of Biotechnology, Birla Institute of Technology and Science Pilani, Dubai Campus, Dubai International Academic City, Dubai, United Arab Emirates

**Keywords:** alternative crop, pseudocereals, nutritional value, food security, climate resilience, arid regions, sustainable agriculture, GWAS

## Abstract

Agricultural productivity needs to grow in a sustainable way to eradicate hunger and malnutrition, as outlined in the 2030 Agenda for Sustainable Development (SDGs). The demand for healthy, nutritious food is expected to rise by 50% between 2012 and 2050 as the world’s population grows. Even today, more than 800 million people face chronic hunger, while 2 billion suffer from micronutrient deficiencies. These challenges are further intensified by climate change stressors. Around 90% of the world’s farmland is affected by climate-related stress, which in some areas can cut crop production by as much as 70%. Countries near the equator, particularly arid lands, are evenly affected, where food security and sustainability are increasingly threatened by rising global food demand and worsening climatic conditions. Relying only on traditional staple crops like rice, wheat, and maize is not enough, and there is a need to explore alternative crops which are climate resilient and could contribute to food security. This review focuses on pseudocereals—crops such as amaranth, quinoa, and buckwheat. These are not true cereals but are rich in nutrients and can survive in difficult environments such as during drought, in salty soils, and at extreme temperatures. Pseudocereals such as amaranth, quinoa, and buckwheat are non-grass crops with dense nutrients. The review covers how pseudocereals can help with food security, improve health, and be used in industry. Some studies have shown that the bioavailability of pseudocereals can be increased by various processing techniques. However, these crops are mostly grown in their native regions because seeds are hard to get and markets are limited. Pseudocereal production must be expanded globally supported by strategies such as conservation of its wild species, molecular advance techniques, policies, farming practices, and integration of indigenous knowledge. Particularly, in arid regions where traditional crops face many challenges due to harsh climatic conditions and limited water resources, integrating these pseudocereal crops into their agronomy system and commodity markets could serve as a roadmap in achieving sustainable development goals (SDGs). These crops could also help other vulnerable regions around the world that face hunger and poor nutrition.

## Graphical Abstract

**Figure:**
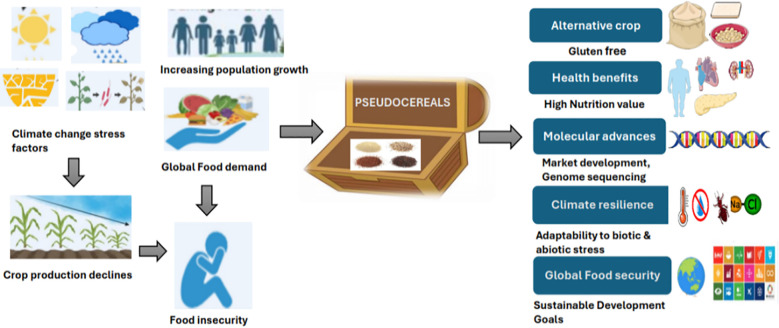
GRAPHICAL ABSTRACT

## Introduction

According to the Sustainable Development Goals (SDGs), hunger and malnutrition must be eradicated by 2030 in a sustainable way by enhancing agricultural productivity—without harming the planet or future generations. Global food demand is projected to rise by approximately 50% between 2012 and 2050, requiring transformative changes in both production and consumption systems (Smith and Gregory, 2013). Despite advances in food systems, over 800 million people remain undernourished, and nearly 2 billion suffer from micronutrient deficiencies. These challenges are compounded by increasingly erratic and extreme weather events, which disproportionately affect marginalized and rural communities. Currently, nearly 90% of global agricultural land is exposed to different abiotic stress such as heat, salinity, nutrient deficiency, drought, pollution, and mechanical stress—that collectively limit crop productivity by up to 70%. The growing intensity of these climate-induced stresses exacerbates socioeconomic vulnerabilities and undermines agro-ecosystem resilience, further threatening food and nutritional security (Diramo Kofa et al., 2024; Begizew, 2021). In arid and semi-arid regions, food insecurity is aggravated by water scarcity, poor soil fertility, and inadequate agricultural practices. These areas are particularly susceptible to climate variability and limited resource availability, necessitating innovative and adaptive agricultural strategies. Despite the identification of over 400,000 plant species globally—of which 30,000 are known to be edible, only 150 crops are cultivated on a significant scale. Alarmingly, just three staple crops—maize, wheat, and rice—account for nearly 60% of daily protein and carbohydrate intake worldwide (Brouns and Shewry, 2022). It is reported that in the next 60 years, the world population will reach up to 10.3 billion (Lam, 2025). To feed this much population, we have to meet the proper food supply with increased productivity and high quality standards. If we will not include additional food varieties other than staple food, we will not be able to combat the upcoming projected food crisis.

Over the past century, approximately 75% of crop diversity has been lost, representing an estimated 300,000 varieties. This erosion of genetic diversity, driven by monocropping and the widespread use of high-yield hybrid crops, has masked local crop varieties and traditional cultivation knowledge, increasing vulnerability to pests, diseases, and environmental stresses. In response, multidisciplinary approaches are being explored to address the multifaceted challenges facing global agriculture—for example, sandponics—a technique that utilizes sand as a sustainable, water-efficient growth medium—has shown promise for food production in arid environments (Nair et al., 2024). Research studies are focusing on genetic engineering and molecular breeding techniques which could make crops grow better and withstand environmental stress factors (Xing and Wang, 2024). Climate-resilient and neglected crops, on the other hand, are proving to be a promising solution to these environmental stresses which could improve nutrition and food security especially in regions with limited resources (Otundo Richard, 2024).

**Figure 1:**
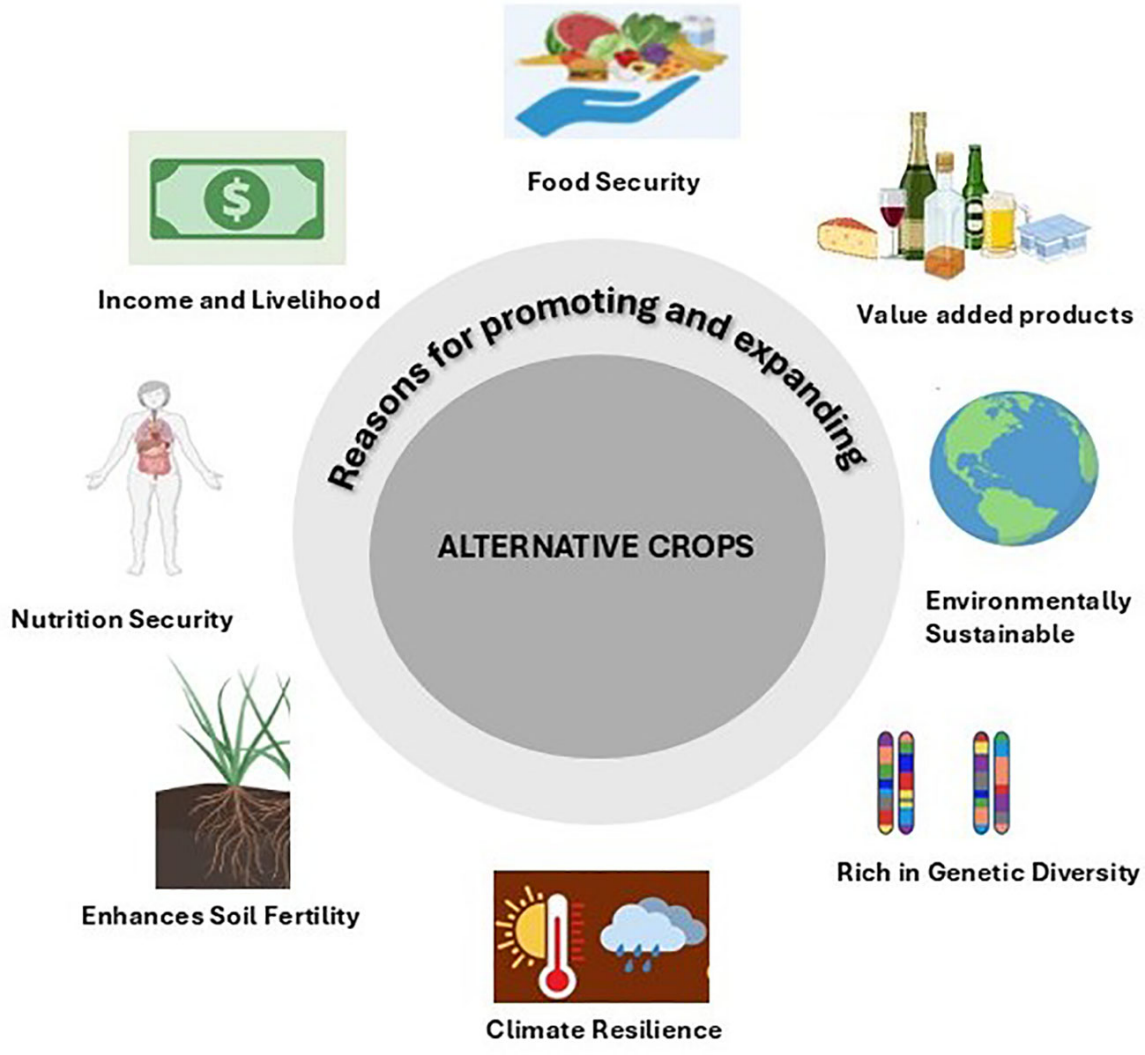
An overview of promoting and expanding alternative crops.

Climate change is growing more complex and causing uneven effects on crop productivity around the world. It has led to a significant loss in some regions such as 13.4% decline in oil palm production—while in others, for example, with soybeans, yields have seen slightly more of approximately 3.5% (Ray et al., 2019). These losses in crop production vary greatly by region: Europe, Southern Africa, and Australia have experienced mostly negative outcomes, whereas Asia, North America, and Latin America show a mix of positive and negative outcomes. Staple crops like rice, wheat, and maize are especially vulnerable in equatorial areas, where rising temperature and water shortage reduce the crop productivity (Farooq et al., 2023). Managing these interconnected issues requires strategies such as diversifying crops, improving water management, and adopting climate-smart farming techniques (Prajapati et al., 2024; Chen et al., 2017). **Figure 1** provides a summary of the key drivers for integrating alternative crops in agriculture.

## Pseudocereals as alternative crops

**Figure 2:**
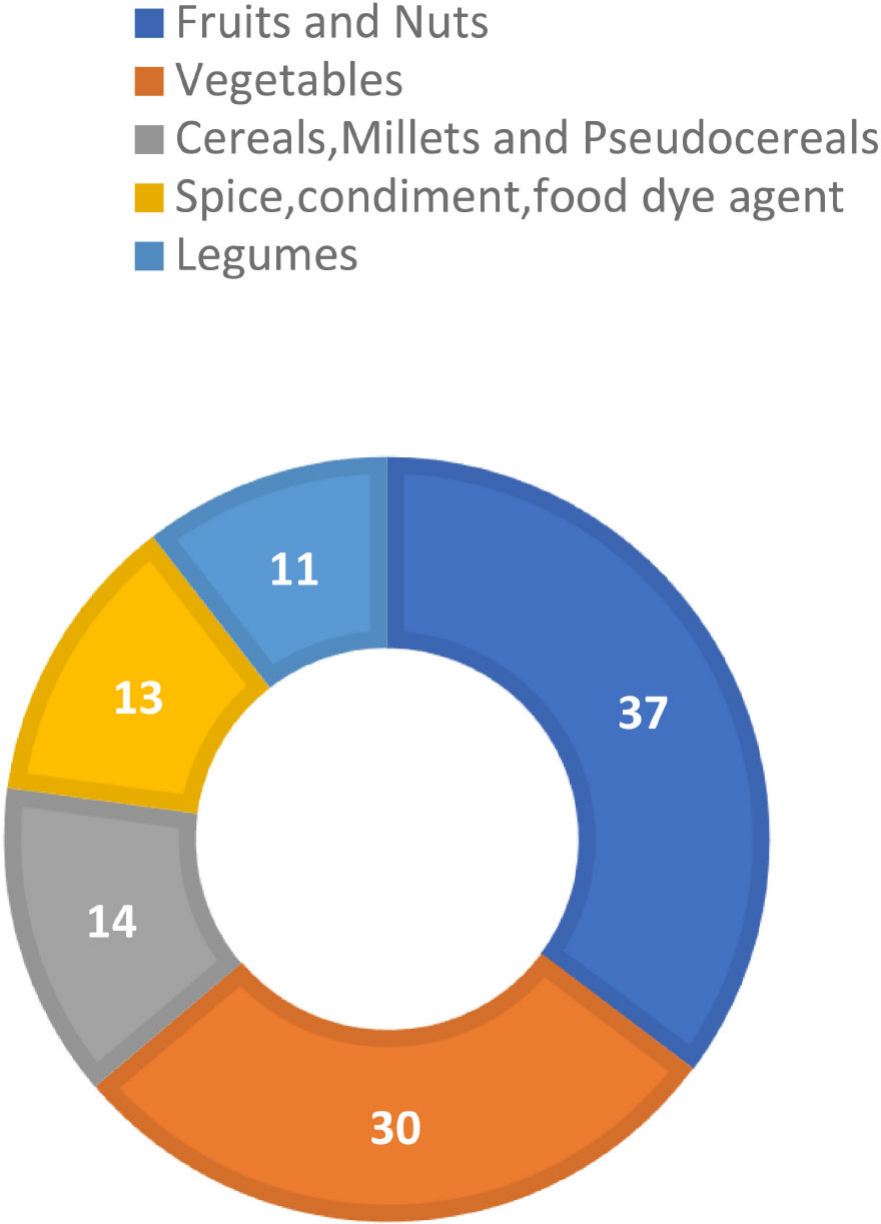
Pseudocereals’ contribution in alternative food groups. (Modified from Pradhan et al., 2021).

Alternative crops, also called orphan or underutilized crops, include many plant species. Despite their potential, they have received little attention from scientists, breeders, and policymakers. The production of these crops is way too less, i.e., approximately 400–500 times, in comparison to staple crops such as rice, wheat, and maize. Still their % compound annual growth rate is 1.5 to 2.1 times than that of cereal crops (Nandan et al., 2024). These non-commodity crops, both domesticated and wild, have immense potential for agriculture. Though historically sidelined for agronomic, economic, or cultural reasons, they are now gaining more attention for their resilience amidst climate change and their ability to resist pests, diseases, and other farming challenges. Researchers worldwide are studying pseudocereals for their role in building sustainable and diverse food systems. The distribution of species in various categories of alternative crops, where the majority proportion accounts for fruits and nuts, is shown in **Figure 2**. The figure depicts that pseudocereals contribute only 14 species, indicating their significant role as alternative crops in different food groups. Naturally gluten-free pseudocereals are rich in amino acids, fatty acids, vitamins, and minerals, supporting better nutrition (Sindhu and Khatkar, 2019; Rao and Poonia, 2023). They can grow in poor soil, making them a sustainable food source in arid regions. Currently, they are mainly grown in native areas, highlighting the need to expand the cultivation worldwide to meet the rising demand (Sindhu and Khatkar, 2019; Nandan et al., 2024).

Quinoa crop has been referred to as “superfood” as it has historical background of over 5,000 years in the agriculture sector, and it is believed to be originated in the Andean region, with highest production in some South Asian countries such as Bolivia and Peru (Fabio and Parraga, 2017). The consumption of quinoa has been reported as maximum in North America, having the highest market share, i.e., 30%. Asia Pacific and Europe are the second and third in terms of the number of consumers and market share, which are 25% and 20%, respectively. It is reported that quinoa consumption has increased exponentially after 2013 (Hunt et al., 2018). The market size of quinoa is expected to increase up to $124.27 at the end of 2025 at a compound annual growth rate (CAGR) of 10.2% (The Business Research Company, 2025). They contain a variety of phytochemicals, which can help in managing obesity, heart diseases, cancers, and diabetes (Singh and Singh, 2016). Quinoa pseudocereal is the preferred diet for celiac patients as it does not contain gluten (Martínez-Villaluenga et al., 2020). Some of the quinoa accessions showed significant performance in terms of grain yield, quality, protein, and dry matter content (Rao and Shahid, 2012).

One of the important essential amino acids, lysine, lacking in other grains, is found abundantly in amaranth pseudocereal (Jagadeeswaran et al., 2022), which makes it an excellent choice in addressing the challenges of global malnutrition and food insecurity (Weerasekara and Waisundara, 2020). Amaranth can be used for cultivation in arid and semi-arid regions (Jagadeeswaran et al., 2022). Recently, amaranth has gained popularity because it can be integrated with modern health-conscious diets (Sharma, 2017). They are cultivated and originated mostly in the various parts of Africa, Central and South America, South-East Asia, and North America (Fabio and Parraga, 2017). A recent report shows that North America has increased consumer demand for amaranth. Apart from this, some parts of Europe such as Germany, Italy, and France and Asia Pacific regions like India, China, and Japan also have high market growth and consumption for amaranth due to increased awareness about its potential benefits (Fortune Business Insights, 2024). According to Maximize Market Research, 2022–29, the CAGR for amaranth is 11.51% (Nandan et al., 2024). In the case of buckwheat, Central Asia and Siberian steppe regions are considered the native place. It is prominently used as staple food in some countries of Western Asia and Eastern Europe (Fabio and Parraga, 2017). It is reported that right now China is the largest producer of buckwheat, with a total production of more than 55%, and its consumption is increasing day by day because of its health benefits. Some European countries such as Russia, Poland, France, and Ukraine are also big names in the buckwheat market as leading producers (Vidaurre-Ruiz et al., 2023). It is reported that the annual cultivation of buckwheat in 2019 was 1,673,478 ha worldwide, along with the production of almost 2,042,401 tons (Graziano et al., 2022). It contains almost all essential amino acids with no gluten and has many nutritional and medicinal properties along with the ability to grow in less fertile soil (Fortune Business Insights, 2024). The anticipated increase in the market of buckwheat industry is expected to reach or grow up to US$33.14 billion by 2034 from US$19.15 billion in 2025, with expected CAGR of approximately 6.3% for the next 10 years (Market Research Future, 2025). These characteristics of pseudocereals contribute to their role in promoting sustainable agriculture.

While quinoa, amaranth, and buckwheat are the most well-known pseudocereals (Bender and Schoenlechner, 2021), other less common varieties exist. These include fonio (*Digitaria exilis* and *D. iburia*), intermediate wheatgrass (*Thinopyrum intermedium*), and wild rice (*Zizania palustris*) (Williams, 1995). The unique chemical, physical, and processing properties of pseudocereals, such as smaller seed kernel size and specific starch structure, distinguish them from traditional cereals. Canihua is mentioned as another important pseudocereal alongside the more common varieties (Bender and Schoenlechner, 2021). Intermediate wheatgrass (*Thinopyrum intermedium*) shows strong potential as a sustainable bread ingredient, with 15% flour substitution yielding optimal loaf volume, texture, and antioxidant properties while enhancing the pigment and color (Williams, 1995). Studies on the germination (24–72 h at 28°C) of *Digitaria exilis* and *Digitaria iburua* significantly enhanced its protein, dietary fiber, amino acids, minerals, resistant starch, phenolics, and antioxidant activity while reducing antinutritional factors. It improved water and oil absorption capacity and slightly altered pasting and thermal properties, with a decrease in bulk density. Germination time, rather than variety, was the main factor influencing these changes, highlighting its potential to produce nutritionally enhanced fonio for novel food applications (Bassey et al., 2023). Studies on genomic analysis of 265 accessions revealed that white (*Digitaria exilis*) and black (*D. iburua*) fonio underwent independent domestications without gene flow, with cultivation expanding in the early Common Era and later declining due to social and agricultural shifts, including the slave trade and crop introductions, providing valuable resources for conserving these climate-resilient cereals (Kaczmarek et al., 2025). Another study on cultivated northern wild rice (*Zizania palustris*) showed that it is a high-value crop primarily grown in Minnesota and California, with domestication starting ~60 years ago to meet rising demands. Breeding has focused on seed retention, yield, and size, but progress is limited by its unique seed physiology and annual growth cycle. Recent advances include a reference genome and improved genotyping methods, enabling comparative genomics with *Oryza sativa* to identify key domestication traits. Given its ecological, cultural, and agricultural importance in the Great Lakes, breeding programs emphasize the conservation of natural stands and inclusion of diverse stakeholders (McGilp et al., 2023).

## Adaptability of pseudocereals

Research studies on pseudocereals have investigated adaptability to marginal soils and varied climatic conditions for improving food security, particularly in regions facing environmental and agricultural challenges. Studies have shown that quinoa and amaranth could thrive in high-altitude areas exceeding 3,000 meters above sea level. When compared to traditional crops, pseudocereals are remarkably resilient to climate change stress factors. Quinoa is an extremophile, able to survive in salty and dry environments (Pizzio, 2022), while amaranth tolerates heat thanks to special heat shock proteins (Goel et al., 2023). These characteristics of pseudocereals make then valuable assets toward more sustainable and climate-resilient agriculture (Nagaraja et al., 2024; Hlásná Cepková et al., 2022). A research study was conducted in the Arabian Peninsula region where the soil fertility and water resources are limited. Buckwheat can adapt to extreme conditions and has a shorter cultivation period. Even though the crop has been underutilized, in some regions it remains an important source as a functional ingredient in health-conscious and native foods (Potkule et al., 2021; Mahata, 2018; Sonawane et al., 2023; Noreen et al., 2020). One of the research studies on quinoa variety that has been investigated for its effect on different salinity levels showed a significant increase in protein levels, suggesting that the crop may possess genetic traits related to salinity stress tolerance (Derbali et al., 2021). Another study showed that growing quinoa with pomegranate in an agroforestry system could help manage soil salinity and improve land use efficiency (Abidi et al., 2024).

**Figure 3:**
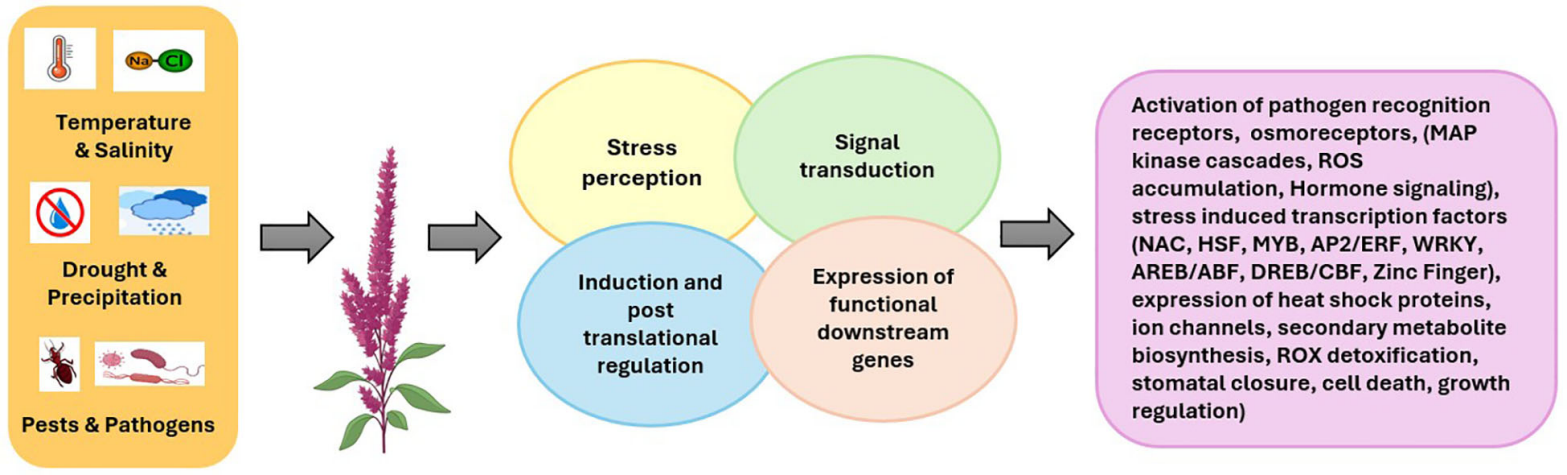
Pseudocereals’ stress resistance/tolerance mechanisms to abiotic and biotic stress factors.

Furthermore, quinoa and amaranth are widely recognized as climate-resilient crops that can withstand drought and extreme temperature conditions due to less leaf surface area, wax-coated leaves, and deep root system. Studies on pathogen resistance in pseudocereals using a model plant *Eutrema salsugineum* showed enhanced pathogen resistance against *Pseudomonas syringae*. This resistance is due to the activation of *PR1*, a defense-related gene (Yeo et al., 2015). These antimicrobial properties could be a valuable tool for breeding pseudocereals in developing climate-resilient cultivars (Banoth et al., 2024; Madhu et al., 2023). In Tartary buckwheat, research studies have highlighted its resistance to abiotic stresses such as aluminum toxicity, cold temperatures, and drought because of the production of rutin. Recent research has identified a new gene, *FtbZIP5*, from Tartary buckwheat showing a significant role in drought and salinity tolerance when the gene was introduced into transgenic *Arabidopsis* plants. *FtbZIP5* gene triggers ABA-related signaling pathways specifically causing a strong expression of several key stress-responsive genes such as *RD29A*, *RD29B*, *RAB18*, *RD26*, *RD20*, and *COR15* (Li et al., 2020). The various mechanisms by which pseudocereals respond to stressors—such as drought, salinity, pathogens, and pests—are illustrated in **Figure 3**.

## Bioavailability and health benefits of pseudocereals

The demand for health-focused products is rising due to growing consumer awareness of their dietary benefits. The current research emphasizes the development of innovative gluten-free products using pseudocereals, alongside efforts to enhance their functionality through processing aids like starches, hydrocolloids, and techniques such as extrusion (Woomer and Adedeji, 2020; Martínez-Villaluenga et al., 2020). The global gluten-free market is expanding rapidly, with sales reaching $6.47 billion in 2023. Moreover, health-conscious consumers are actively seeking foods that help lower the risk of illnesses like cancer, diabetes, and heart diseases. Rich in nutrients and bioactive compounds as mentioned in **Table 1**, pseudocereals align well with these preferences (Martínez-Villaluenga et al., 2020; Kaur, 2023; Thakur et al., 2021). Rising cases of celiac disease and gluten sensitivity have boosted the demand for gluten-free options. As naturally gluten-free grains, pseudocereals are ideal for these diets (Rollán et al., 2019; Wanniarachchi et al., 2023; Szűcs, 2023). However, their commercialization is still limited due to processing challenges and low consumer acceptance (Xu et al., 2020; Alvarez-Jubete et al., 2010; Woomer and Adedeji, 2020). To make pseudocereals healthier and easier to digest, a variety of processing methods—like soaking, cooking, fermentation, and even microwave or irradiation techniques—are commonly used. Heat treatments are widely used for things like sterilization and enhancing flavor. Overall, both traditional methods like baking and milling and more advanced ones like enzyme-based processing play an important role in shaping the nutritional value of pseudocereals (Langyan et al., 2024).

**Table 1:** Bioavailability of pseudocereals.

Nutritional parameters	Quinoa (*Chenopodium quinoa*)	Amaranth (*Amaranth* spp.)	Buckwheat (*Fagopyrum esculentum*)
Proximate composition Crude protein	13.11	14.59	13.25
Total fat Dietary fiber Total Insoluble Soluble Carbohydrate	5.50 14.66 10.21 4.26 53.65	5.74 7.02 5.76 1.26 59.98	3.40 10.00 - - 71.50
Mineral composition (mg/100 g) Copper (Cu) Calcium (Ca) Magnesium (Mg) Iron (Fe) Manganese (Mn) Potassium (K) Phosphorus (P) Zinc (Zn) Sodium (Na)	198 0.48 7.51 119 1.77 212 474 4.50 3.31	181 0.81 9.33 325 5.29 374 433 2.70 2.66	18 1.10 2.20 231 1.30 347 460 1.00 2.40
Vitamins α-Ergocalciferol (vitamin D) (µg) α-Tocopherol (vitamin E) (mg) Phylloquinones (vitamin K1) (µg) Thiamine (vitamin B_1)_ (mg) Riboflavin (vitamin B_2_) (mg) Niacin (vitamin B_3_) (mg) Pantothenic acid (vitamin B_5_) (mg) Vitamin B_6_ (mg) Biotin (vitamin B_7_) (µg) Folates (vitamin B_9_) (µg)	- 2.08 2.00 0.83 0.22 1.70 0.62 0.21 0.62 1.73	0.04 1.92 - 0.04 0.04 0.45 0.24 0.50 1.92 27.44	- 0.32 7.00 0.42 0.19 6.15 0.44 0.58 - 54.00
Amino acids (g/100 g protein) Arginine (ARG) Alanine (ALA) Glutamic acid (GLU) Aspartic acid (ASP) Glycine (GLY) Proline (PRO) Serine (SER) Tyrosine (TYR) Histidine (HIS) Isoleucine (ILE) Leucine (LEU) Lysine (LYS) Methionine (MET) Cystine (CYS) Phenylalanine (PHE) Threonine (THR) Tryptophan (TRP) Valine (VAL)	4.35 7.85 8.40 13.75 4.80 5.67 4.56 1.98 2.98 3.75 6.08 5.55 2.24 1.85 4.35 3.01 1.25 4.55	4.26 7.77 12.57 16.12 8.50 3.76 7.79 2.85 1.86 2.82 4.83 5.45 1.86 1.60 3.98 3.02 1.05 4.34	4.50 9.70 11.30 18.60 6.30 3.80 4.70 2.10 2.70 3.80 6.40 6.10 2.50 1.60 4.80 3.90 2.00 4.70

Hyphens (-) indicate values that were either below the limit of detection or unreported. The data were compiled from multiple sources, including Dayakar et al. (2017); Gopalan et al. (1989); Johnson and Croissant (1985); Longvah (2017); Pomeranz and Robbins (1972); Ikeda and Kishida (1993), and the USDA National Nutrient Database for Standard Reference (accessed October 28, 2019).

Pseudocereals are rich in antioxidants and soluble fiber which help regulate blood sugar, improve digestion, and lower blood cholesterol levels, supporting heart health. The fiber content is very similar to that in fruits and vegetables. This fiber, along with other beneficial compounds, has been associated with antioxidant and anticancer effects, boosting the immune system. These superior health-promoting properties make pseudocereals a promising choice in developing functional foods (Zhu, 2020). Researchers have identified six bioactive peptides in amaranth that may strongly inhibit angiotensin-converting enzyme (ACE) activity, suggesting a role in controlling blood pressure and supporting cardiovascular health (Toimbayeva et al., 2025). Pseudocereals such as Tartary buckwheat, amaranth, and quinoa offer a wide range of health benefits due to their rich bioactive compounds. Tartary buckwheat has been shown to lower plasma cholesterol, reduce inflammation, inhibit cell proliferation, and induce apoptosis, primarily through its proteins and polysaccharides. Amaranth contributes to reducing blood cholesterol levels and exhibits antioxidant, antimicrobial, anti-inflammatory, and hepatoprotective properties, with its protein hydrolysates and peptides playing a key role. It also shows promise in osteoporosis treatment. Quinoa offers antioxidant effects, promotes gut health, and reduces inflammation in gut cells due to components such as chenopodin protein (Kaur, 2023). Studies on lactic acid fermentation of protein-rich amaranth flour with probiotic LAB strains *Lacticaseibacillus rhamnosus* MIUG BL38 and *Lactiplantibacillus pentosus* MIUG BL24 showed enhanced antioxidant activity and increased phenolic content—particularly epigallocatechin—and demonstrated potential for developing gluten-free, tribiotic-enriched functional foods (Souare et al., 2025).

Quinoa saponins have been suggested to possess immunoadjuvant activity, as shown in studies with mice immunized with ovalbumin, where both humoral and cellular immune responses were enhanced (Verza et al., 2012). In another study, oat- and Tartary buckwheat-based diets were fed to hypercholesterolemic hamsters. The results indicated that these foods could lower serum lipid levels by reducing cholesterol absorption in the liver and significantly promoting lipid excretion in feces. They also boosted short-chain fatty acid production, which helped regulate the gut microbiota and contributed to the effective management of hypercholesterolemia (Sun et al., 2019). Amaranth oil has been linked to improved cardiovascular health, with participants reporting fewer symptoms such as headaches, weakness, and exercise-induced exhaustion. Remarkably, the cardiac rhythms in 40%–50% of participants returned to normal during the study (Thakur et al., 2021). In overweight women, consuming 25 g of quinoa flakes daily for 4 weeks significantly reduced blood triglycerides, total cholesterol, and low-density lipoprotein (LDL) cholesterol in both prospective and double-blind intervention trials (De Carvalho et al., 2014). Some species of amaranth also show anti-cancer potential. Compounds in *Amaranthus tricolor* have been found to inhibit tumor cell proliferation, while proteins from *Amaranthus hypochondriacus* seeds contain peptides linked to cancer-preventive effects. An amaranth lunasin-like peptide was shown to inhibit H3 and H4 histone acetylation in HeLa cells, with the effect being dose-dependent. This epigenetic mechanism, similar to that found in soybean and barley, may explain lunasin’s ability to help prevent cancer (Huerta-Ocampo and de la Rosa, 2011). Quinoa also demonstrates antioxidant properties. In animals fed a high-fructose diet to induce oxidative metabolic stress, quinoa consumption increased the activity of key antioxidant enzymes and reduced lipid peroxidation in plasma, red blood cells, and multiple organs, including the heart, kidney, liver, and brain (Pasko et al., 2010). In terms of cancer-related effects, buckwheat polysaccharides did not directly inhibit the growth of human PC-3 prostate cancer cells but instead reduced their proliferation by stimulating the release of anti-inflammatory biomarkers (Lin and Lin, 2016). Similarly, in rats with induced tumors, buckwheat protein was found to protect against colon cancer by inhibiting cell proliferation (Tomotake et al., 2006). Buckwheat has also been shown to support gut health. In experimental rat models, buckwheat-based diets increased the populations of aerobic mesophilic and lactic acid bacteria, particularly *Lactobacillus plantarum* and *Bifidobacterium* spp (Préstamo et al., 2003). Quinoa has even been used to create a symbiotic beverage that extended the fermentation period and enhanced the survival of *Lactobacillus casei* LC-1 (Bianchi et al., 2015). For individuals with diabetes, buckwheat administration—both in chronic and acute cases—has been shown to improve metabolic and cardiovascular markers (Stringer et al., 2013). Amaranth protein likewise improved glucose tolerance and plasma insulin levels in a streptozotocin (STZ)-induced diabetes model. In diabetic rats, amaranth oil and grain supplementation prevented increases in total cholesterol, triglycerides, and VLDL while also reducing hyperglycemia caused by STZ by 77% and 81%, respectively (Martínez-Villaluenga et al., 2020). These findings underscore the potential of pseudocereals as functional foods for preventing and managing various health conditions.

## Pseudocereals in food security

In today’s market-driven food systems, pseudocereals are gaining attention as valuable ingredients. Processing methods like lactic acid fermentation can improve their nutritional and functional qualities. After processing, pseudocereals are used in baked goods, fermented drinks, and extruded snacks (Alencar and de Carvalho Oliveira, 2019; Graziano et al., 2022; Martínez-Villaluenga et al., 2020). Traditional cereal-based foods can be blended with pseudocereals which will be an effective way to enhance their nutritional value. One study tested adding 50% refined or whole-meal quinoa, amaranth, and buckwheat flours into water biscuits (WB) to evaluate the antioxidant capacity and heat resistance. Buckwheat had the highest tocol content (86.2 mg/kg), einkorn had the highest carotenoids (5.6 mg/kg), and buckwheat and quinoa had the most conjugated phenolics (230.2 and 218.6 mg/kg, respectively). The WB with pseudocereals showed better antioxidant profiles and less heat damage compared to 100% einkorn or bread wheat WB, although lysine loss was higher. They also had a more balanced amino acid profile (Estivi et al., 2022). Processing techniques like sprouting, cooking, and fermentation offer more opportunities for health-focused products. A study on quinoa (var. Tunkahuan) and amaranth (var. Alegría) found that germination and 24-h fermentation increased polyphenols and flavonoids, while fermentation with *Lactobacillus plantarum* greatly boosted antioxidant activity.

Germinated seeds exhibited higher macro- and microelement content compared to raw seeds. Tests using the *S. cerevisiae* D7 strain confirmed that seed and germinated seed extracts had no genotoxic effects and protect cells from damage by reactive oxygen species (ROS). These findings suggest that germinated seeds and fermented products from these varieties are highly suitable for inclusion in diets and dietary supplements (Vento et al., 2024). **Table 2** summarizes the different products made using pseudocereals and their applications. Dehulled buckwheat seeds are rich in essential nutrients and bioactive compounds. Studies showed that buckwheat flour (30%) used to make bread had good sensory and baking qualities. Similarly, pasta can be enriched with proteins, minerals, and rutin by using buckwheat flour without affecting its cooking and sensory characteristics (Marti et al., 2011). Amaranth is high in protein, while starch, fat, fiber, and mineral contents are similar in quinoa and amaranth. Buckwheat has more starch, moderate protein, and fewer fats, fibers, and minerals, but with the highest phenolic content. All three pseudocereals are rich in phosphorus, potassium, and magnesium. Polysaccharides in pseudocereal cell walls were examined for their structural and functional traits, which is comparable to those typically found in fruits and vegetables, suggesting that pseudocereals might offer comparable or even more health benefits when used in the formulation of food products (De Bock et al., 2022). Quinoa, amaranth, and buckwheat are valued for their proteins, fiber, bioactive compounds, and folic acid (Gorinstein et al., 2002; Das and Das, 2016; Schoenlechner et al., 2010). Their strong nutritional profiles make them important for food and nutrition security, with quinoa and amaranth—often called “nutri-cereals”—showing great potential in production, consumption, and trade.

**Table 2:** Industrial applications of pseudocereals.

Products	Applications	References
Gluten-free bakery products	Amaranth improves the nutritional quality of food that lacks gluten	Gambus et al., 2010
	Bread made from buckwheat had significantly higher mineral composition than wheat breads	Islam et al., 2016
	10% of amaranth flour with cheese bread could score 6.8 out of 9 in hedonic scale showed enhanced iron and dietary fiber content	Lemos et al., 2012
	Gluten-free bread with 60:40 ratio of popped and raw amaranth flour was found to increase in volume and produced consistent crumb	de la Barca et al., 2010
Noodles and pasta	Buckwheat does not contain gluten ingredient for improving texture of noodles, as it enhanced firmness and reduced cooking loss For quinoa pasta, emulsifiers are added to enhance its quality Corn flour and quinoa flour (5%–15%) were investigated in making gluten-free spaghetti. Quinoa and rice flour, gluten-free blends have been used to make tasty macaroni and pasta	Borges et al., 2003
Beverages	Beers brewed with buckwheat malt showed similar physicochemical properties to traditional wheat-based beers, including pH, amino acid content, fermentability, and alcohol content. Also delivered satisfying sensory qualities, with a pleasant aroma, balanced taste, and appropriate bitterness Gluten-free, bottom-fermented beers made from buckwheat and quinoa malts have been successfully brewed, showing viscosity and pH levels much like those found in traditional barley beers Buckwheat’s bioactive components are suitable for tea production	Phiarais et al., 2010 Qin et al., 2013
Animal fodder	Buckwheat plant residues can be used as animal feed Quinoa plant is rich in minerals and plant residues can be utilized as animal feed Amaranth can be used to reduce cholesterol level in animals	Leiber, 2016 Zulkadir and İdikut, 2021 Peiretti, 2018
Granolas and breakfast cereals	Granolas made with quinoa, amaranth, or linseed were well received for their taste and offered strong nutritional and physico-chemical benefits. Had low water activity, which helped extend shelf life, while still providing high nutritional value and appealing flavor and texture Quinoa and amaranth can serve as a substitute for rice and breakfast porridge or as a base in infant food formulation Quinoa and cranberry extract as breakfast cereal showed highest anthocyanin and phenolic content	Schoenlechner, 2017 Jancurová et al., 2009 Srujana et al., 2019

## Recent molecular advances in pseudocereals

In recent years, molecular studies have been accelerated due to advancements in next-generation and transcriptome sequencing analysis. This has made the study of molecular markers and the application of molecular breeding very easy. Genetic improvements can be further pursued based on available knowledge about amaranth (*A. hypochondriacus*), with 466-Mb genome and 24,829 protein-coding genes (Sunil et al., 2014); quinoa, with a 1.5-Gb genome size and 54,438 annotated genes (Zou et al., 2017); and buckwheat (*F. esculentum*), with a 1.12-Gb size and 35,186 protein-coding genes (BGDB; http://buckwheat.kazusa.or.jp) (Yasui et al., 2016). Three different research groups (Yasui et al., 2016; Jarvis et al., 2017, and Zou et al., 2017) have performed next-generation sequencing for the sequencing of the quinoa genome. In quinoa, molecular markers like SSR and SNP and insertion/deletion markers have been identified for 11 accessions (Zhang et al., 2017). Drought tolerance genes have been found in two genotypes (Raney, 2012) and drought-induced genes and pathways in the Chilean genotype R49 (Morales et al., 2017). Quinoa also has a higher level of lysine and more vitamins E and B than many cereals (Zou et al., 2017). Research on the drought-tolerant quinoa genotype “Dianli 129” found 38,670 genes and 142 pathways. Changes in specific genes and metabolites helped maintain flavonoid, starch, and sucrose metabolism—key to drought stress resistance (Huan et al., 2022).

A study of quinoa germplasm from eight countries used the iPBS-retrotransposon marker system with 11 highly polymorphic primers to assess genetic diversity. It provided data on polymorphism percentage, mean PIC, effective alleles, Shannon’s index, and gene diversity (Barut et al., 2020). A recent study by Rahman et al. (2024) analyzed quinoa accessions for agronomic and biochemical traits using next-generation sequencing. They found nine marker–trait associations for saponin content across eight chromosomes, offering tools for marker-assisted selection to develop sweeter, higher-yield quinoa. Xiao-Lin et al. (2022) identified 13 SnRK2 genes, which play key roles in ABA signaling and stress responses. Tariq et al. (2022) confirmed that the CqKCS2B.1 gene helps quinoa tolerate salt stress by regulating suberin biosynthesis, opening possibilities for breeding salt-tolerant varieties.

Two cultivars of quinoa, named Dianli-3101 and Dianli-3051, have been studied extensively by Xie et al. (2023) under very high temperature conditions. They found some photosynthetic genes that were downregulated and a large change in differential accumulation for lipids and flavonoids (Xie et al., 2023). Transcriptomics has been key in identifying stress-protective genes in amaranth. Singh et al. (2024) compared the genomes of five amaranth species—*A. hypochondriacus*, *A. cruentus*, *A. palmeri*, *A. hybridus*, and *A. tuberculatus*—identifying 170,477 protein-coding genes, with most repeats being LTRs. They found species-specific SNPs linked to a variation in commercially important genes. Translational and post-translational studies, such as microRNA-guided silencing, along with transcription factors like bHLH, NAC, bZIP, C2H2, Dof, AP2/ERF, WRKY, and MYB, play major roles in stress response.

In buckwheat, Zhang et al. (2021) conducted whole-genome resequencing of 510 germplasms and created a genomic variation databank for Tartary buckwheat. They identified candidate genes, such as FtUFGT3 and FtAP2YT1, linked to flavonoid accumulation and grain weight. Based on these findings, two varieties with different traits were developed through separate domestication events. Zhao et al. (2023) showed that domestication can influence metabolite accumulation. Using mGWAS with EMMAx and FaST-LMM on 567 metabolites, they found 1,253 lead SNPs linked to 398 metabolites—291 related to flavonoids and 171 to phenolic acids. One SNP alone was associated with 128 metabolites, including isovitexin and catechin. Chemical modifications and metabolic pathways can improve metabolite stability and availability, supporting the development of superior varieties.

Wang et al. (2025a) studied mineral and trace element variation in 199 Tartary buckwheat accessions. They found that changes in the promoter region of the FtACA13 gene (an auto-inhibited Ca²^+^-ATPase) are linked to salt tolerance and Na concentration. The GWAS analysis identified 52 genetic loci associated with 10 elements. The FtYPQ1 gene, a vacuolar amino acid transporter, was linked to improved Zn tolerance, while the FtNHX2 protein (a Na^+^/H^+^ exchanger) may play a role in arsenic tolerance, supported by a significant signal locus on chromosome 6. A more complete characterization combining phenotypic, nutritional, biochemical, physiological, and molecular data is needed to develop superior genotypes to combat hunger and ensure food security.

Some latest collections of pseudocereal crops in both national and international gene banks have been characterized based on key morpho-agronomic traits as shown in **Table 3**.

**Table 3:** Molecular advances in pseudocereals.

Pseudocereals	Marker development	Studies on genetic variation and population analysis	Gene expression profiling	Quantitative trait loci (QTL) analysis and gene discovery	Whole-genome sequencing	References
Amaranth	Plant material from six *Amaranthus* species were gathered from eight geographic regions of Indo-Gangetic plains, and unique SCAR markers were developed (*A. caudatus, A. cruentus*, *A. gangeticus*, *A. hypochondriacus*, *A. paniculatus*, and *A. viridis*)	Genetic analysis revealed that *A. hypochondriacus* and *A. caudatus* are closely related, as 313 accessions with 0.75% genomic overlap, and were grouped into two distinct genetic lineages Gene-specific primers for SSSI and GBSSI genes were employed to distinguish *A. caudatus* and *A. hypochondriacus*; 22 detected alleles showed an average of 0.657 polymorphism information content, reflecting diversity. *A. powellii* and *A. retroflexus* exhibited the highest SNPs, while *A. quitensis* and *A. caudatus* displayed very close genetic relationships. Genetic diversity was observed between edible and wild amaranth species	Identified 8,260 homologous sequences with *A. tuberculatus* and 1,971 stress-responsive genes. Revealed differentially expressed proteins and transcripts involved in stress defense and signaling pathways. Upregulated transcription factors like DOF1 and MIF1 were associated with stress adaptation and growth regulation. Downregulated genes were linked to cell differentiation and secondary metabolism	Identified AhDODA-1, AhDODA-2, AhcDOPA5-GT, and AhB5-GT genes for betanin biosynthesis. AhNF-YC: Linked with stress resistance and growth. Ah24: A newly identified stress-responsive gene from *Amaranthus cruentus* roots has been associated with reactions to salt stress, herbivore attack, and exposure to methyl jasmonate. In addition, ERF and Dof transcription factors linked to stress were found to play roles in the plant’s response to salt, drought, and signaling molecules like jasmonic acid, salicylic acid, and abscisic acid (ABA)	The transcriptome of *A. hypochondriacus* revealed independent C4 evolution. The grain amaranth genome (377 Mb, 3,518 scaffolds) included 23,059 protein-coding genes, with 48% consisting of repeat sequences. A chromosome-scale assembly (403.9 Mb) constructed with Hi-C chromatin contact maps and PacBio long reads scaffolded 98% of the genome into 16 chromosomes Domestication studies identified a MYB-like transcription factor as a potential regulator of seed coat color variation. Three independent domestication events from a single wild ancestor were observed, with the conversion of dark to white seed coats linked to this process	Wu and Blair, 2017; Casique-Arroyo et al., 2014; Palmeros-Suárez et al., 2015, Julio et al., 2015, 2016; Sunil et al., 2014; Clouse et al., 2016; Lightfoot et al., 2017; Stetter et al., 2020
Buckwheat	AFLP and pooled DNA mapping approaches identified genetic markers associated with *Sht1* allelic site in a cross between non-brittle and brittle buckwheat lines. Microsatellite variability in common buckwheat. SSR marker development in Tartary buckwheat	Identified high levels of genetic variation within cultivars and populations along with 3 RAPD markers. 19 Japanese varieties using 5 microsatellite loci. Reported 86.5% polymorphism in 79 Tartary buckwheat accessions using AFLP markers. Analyzed 179 common buckwheat accessions with SSR markers. GBS in buckwheat revealed more nucleotide diversity (0.0065). RAPD and AFLP markers explored genetic relationships among wild and domesticated varieties	RNA-seq of filling stage seeds. Key genes in storage proteins, flavonoid biosynthesis, transcription factors identified. RNA-seq revealed aluminum-responsive genes involved in cell wall defense and oxidative stress. Identified numerous drought-responsive genes through transcriptomic data analysis	Two dominant genes are identified for seed shattering. QTLs identified for photoperiod sensitivity. High expression of genes for flavonoid and rutin biosynthesis identified. *FtGBSSI* gene in Tartary buckwheat, crucial for amylose synthesis isolated and characterized. Identified AI-responsive genes, abiotic stress genes, auxin-signaling genes. Isolated *FaesAP3*, a MADS-box gene, identified 65 MADS-box genes, Identified *FePG1* associated with heteromorphic self-incompatibility	Produced a draft assembly with 387,594 scaffolds using next-generation sequencing. Combined multiple sequencing approaches, including Illumina short reads, SMRT long reads, Hi-C sequencing, and BioNano genome maps. Annotated 33,366 protein-coding genes, offering a comprehensive resource for functional genomics and breeding programs	Hou et al., 2016; Shi et al., 2017; Mizuno and Yasui, 2019; Gao et al., 2017; Yao et al., 2017; Zhu et al., 2015; Wu et al., 2019; Thiyagarajan et al., 2016; Wang et al., 2014; Yokosho et al., 2014; He et al., 2019; Liu et al., 2018; Fang et al., 2014; Liu et al., 2019; Takeshima et al., 2019; Yasui et al., 2016; Zhang et al., 2017
Quinoa	A linkage map was constructed for a recombinant inbred line (RIL) using 216 polymorphic SSR markers. Morphological characteristic variations together with chloroplast rbcL and matK gene sequences were assessed across 19 *Chenopodium* accessions. Also, the first *C. quinoa*-specific SNP primer, R1RQ-AFR, was identified based on rbcL sequences	Studied 19 accessions of *Chenopodium* using 33 RAPD primers, characterized Chilean and South American quinoa accessions with SSR markers, developed 511 SNP assays and InDels, racked quinoa diversity over 18 centuries with SSR markers, analyzed 121 quinoa accessions, used RAPD and ISSR markers	Identified 20,337 unique transcripts and 462 putative drought- and abiotic-stress-related gene products. Used RNA-Seq to study Groundnut Chlorotic Fan-spot Tospo virus-infected leaves. Performed RNA-Seq across six inflorescence developmental stages	Characterized homologous loci in salt tolerance and found differential expression of genes in shoots and roots under salt stress. Identified 90 NAC transcription factors. Provided two 11S genes with cDNA and genomic sequences. Isolated Ty3-retrotransposon and Ty1-copia retrotransposons. Identified unique specific genes (459) expressed in flowers, meristems and CqAmaSy1 (quinoa amaranthin synthetase 1) gene	Published a draft genome sequence of quinoa, consisting of 25k scaffolds, totaling 1 Gbp genomic size N50 contig length of 86 kbp. Superior quality genome draft was generated, comprising 64.5% repeated sequences including 54,438 genes for protein-coding and 192 microRNA genes	Devi and Chrungoo, 2017; Maughan et al., 2012; Zhang et al., 2017; Winkel et al., 2018; Saad-Allah and Youssef, 2018; Raney et al., 2014; Chou et al., 2017; Wu et al., 2019; Ruiz-Carrasco et al., 2011; Li et al., 2019; Kolano et al., 2013; Golicz et al., 2019; Imamura et al., 2019; Yasui et al., 2016; Zou et al., 2017; Jarvis et al., 2017

Plant databases provide open access to genomic resources for breeders and researchers. The availability of extensive data from genetic markers, complete genetic sequences, GWAS studies, different types of ionome, metabolomics, and transcriptomics studies of millets and other crops like pseudocereals could serve as a valuable insight to researchers that can greatly support efforts to improve medicinal properties, disease resistance, climatic resilience, food innovations, precision breeding, and agriculture sustainability.

## Pseudocereals: challenges and strategies

Climate change in arid regions causes unpredictable weather affecting ecosystems, lowering agricultural productivity, and giving immense stress on traditional crops that have no tolerance to extremes. As a result, valuable plant biodiversity and traditional knowledge about resilient, indigenous crops have been lost. Soil erosion, desertification, and habitat loss are increasing along with pollution exacerbating the condition (Abebaw, 2025). Pseudocereals show promising solutions for these stress factors as the need to find sustainable, drought-resistant, and climate-smart crops increases. These underutilized crops are unique to grow in adverse conditions, but their potential remains untapped. Key challenges are the changing climate and outdated farming practices which are not suitable for arid environments (Bekkering and Tian, 2019). Over-dependence on high water demanding crops, combined with the use of excessive fertilizers, has severely degraded the soil (Wang et al., 2025b). Pseudocereals, with their ability to thrive in diverse soil types and dry climates, offer a sustainable alternative. However, progress should be made on optimizing cultivation methods and crop management technologies based on plant species. Even though alternatives like quinoa or amaranth could offer better yields and nutrition in many arid regions, farmers are still hesitant to try these crops. On the other hand, the growing interest in healthy and sustainable diets presents an opportunity for the wider adoption of pseudocereals—but only if more people become aware of their nutritional benefits and culinary versatility. Nevertheless, their broader consumption is hindered by factors such as the presence of anti-nutritional compounds and a naturally bitter taste, which often necessitate additional processing and, in turn, raise production costs. Furthermore, pseudocereals are unlikely to fully replace true cereals because of certain organoleptic and technological limitations (Graziano et al., 2022). Their cultivation remains largely confined to native regions, which raises concerns about resource exploitation and the socio-economic wellbeing of smallholder farmers (Graziano et al., 2022; Nandan et al., 2024). Processing techniques such as fermentation and germination have shown promise in improving nutritional value and nutrient bioavailability (Henrion et al., 2020), positioning pseudocereals as valuable alternatives to enhance dietary diversity and support better health outcomes.

Migration of farmers to urban areas has further complicated the issue causing lack of manpower in the agriculture sector (Kalantaryan et al., 2021). In arid regions, the overuse of natural resources continues to threaten the long-term sustainability of agriculture. Main crops such as maize, wheat, and corn have been given more importance, and pseudocereals struggle to compete with those crops economically. Moreover, farmers are skeptical to cultivate pseudocereal crops as it is unfamiliar for them due to lack of awareness about its nutritional value and cultivation methods. There is a lack of policy and supportive frameworks for the cultivation and marketing of pseudocereals (Vidaurre-Ruiz et al., 2023). Pseudocereals remain vulnerable due to limited conservation efforts and funding and poor integration between preservation and sustainable use. Access to diverse germplasms and stronger research are essential to breed varieties suited to arid zones (Bekkering and Tian, 2019). Unlocking the potential of pseudocereals will require coordinated efforts to address environmental, agronomic, social, economic, and political challenges.

**Figure 4:**
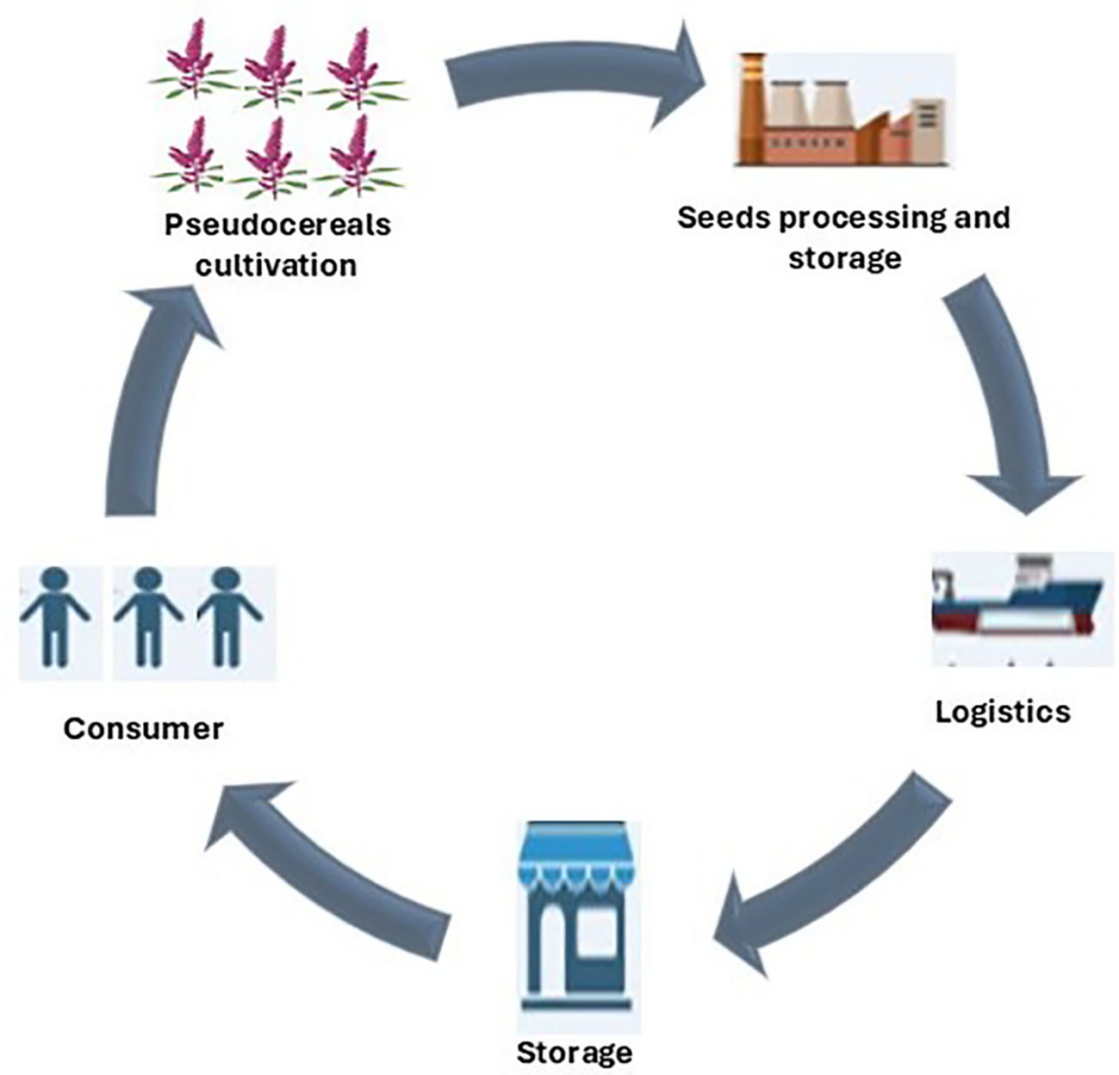
Schematic representation of pseudocereals’ supply chain.

Developing eco-geographic databases for targeted pseudocereal species can help in identifying its ideal growing areas and conditions. Furthermore, building comprehensive databases that track both the nutritional value and social impact of these pseudocereal crops will give policymakers the solid, evidence-based insights they need to make informed decisions (Hoehnel et al., 2022). Mapping suitable ecological zones can reduce competition with major crops while maximizing benefits for people in dry regions. Strengthening local seed systems through collaborative breeding will give farmers access to high-quality seeds. Combining scientific research with traditional knowledge can improve adoption (Shrestha and Gauchan, 2020). Research outcomes, success stories, and lessons learned from farmers, researchers, and community members can be shared to help spread innovation (Zoundj et al., 2024). Another key step in increasing the value chains for pseudocereals is that once they are harvested directly, it should be linked to the consumers (**Figure 4**). This will reduce costs and open up markets for pseudocereals crops. Policymakers can offer incentives and subsidies for pseudocereal crops that encourage crop diversification and support sustainable farming practices (Nandi et al., 2024). Introducing pseudocereals into school feeding programs can build awareness and acceptance among younger generations (Kristjansson et al., 2022). With the right strategies, pseudocereals could play a key role in ensuring food and nutrition security in arid areas.

## Pseudocereal breeding: current trends and future directions

Research and breeding programs on pseudocereal crops remain limited, with amaranth possessing significant genetic diversity. One of the studies on amaranth and quinoa showed that these can respond well to nitrogen fertilization, showing increased grain yields with higher nitrogen input, whereas buckwheat showed minimum to no response (Kaul et al., 2005). In Mexico, traditional farming systems like Milpa and Mogote are used for pseudocereal cultivation. These intercropping systems combine crops like corn, beans, squash, and pseudocereals, offering multiple benefits such as higher yield and protein content and thus contributing to food security and boosting the farmers’ income (Torres et al., 2007). Few studies focus on breeding high-yield, stress-tolerant pseudocereals, and modern tools like CRISPR/Cas and RNA interference are rarely applied (Anuradha et al., 2023; Vats et al., 2023). The conservation of crop wild relatives (CWRs) of pseudocereals is also lacking in gene banks. Expanding molecular research by applying genomic tools, whole-genome sequencing, and modern breeding techniques through an interdisciplinary approach is essential to unlock pseudocereals’ potential for food and nutrition security (Arya et al., 2021; Thakur et al., 2021; Curti et al., 2017; Bekkering and Tian, 2019).

**Figure 5:**
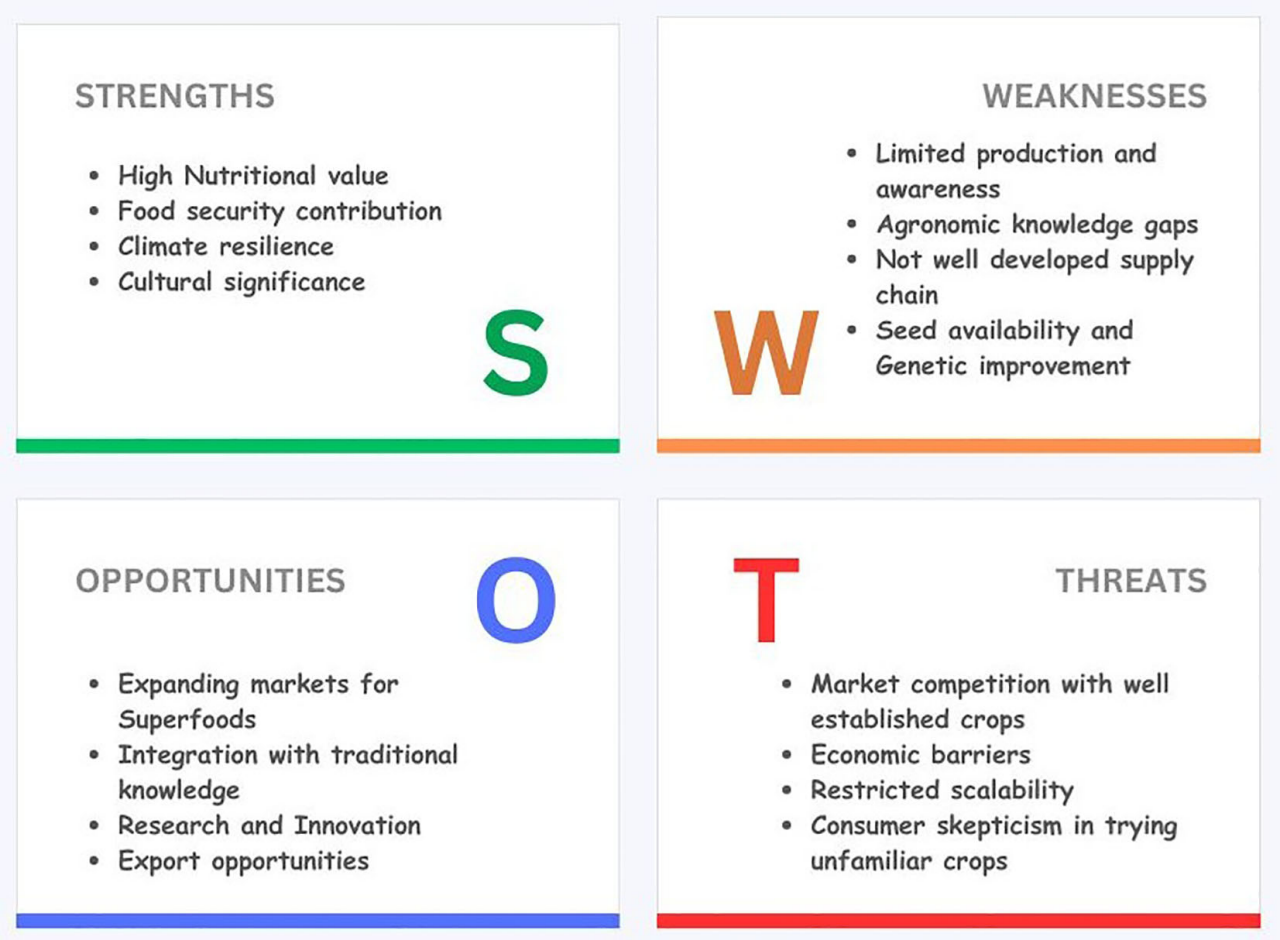
SWOT analysis of pseudocereals.

Despite their nutritional and functional benefits, pseudocereals often face consumer acceptance barriers due to limited awareness of their health-promoting properties, misconceptions about taste and cooking methods, perceived complexity in preparation, and higher prices compared to staple cereals (Bender and Schoenlechner, 2021; Vidaurre-Ruiz et al., 2023). In some markets, the relatively low availability of processed or ready-to-use pseudocereal products further limits adoption. To address these challenges, several government agencies, research institutions, and public health bodies have implemented strategies to increase their popularity. The International Center for Biosaline Agriculture (ICBA) has undertaken extensive research on quinoa and other pseudocereals for saline and marginal environments, supporting farmer adoption in the Middle East, North Africa, and Central Asia. In Mexico, the traditional *milpa* intercropping system, which integrates quinoa and amaranth with maize and beans, demonstrates a sustainable, culturally embedded approach to promoting pseudocereal consumption. In India, the Indian Council of Agricultural Research (ICAR) has conducted breeding and agronomic trials on amaranth, buckwheat, and quinoa to improve yield, stress tolerance, and market potential. Alongside these efforts, policy measures include targeted awareness campaigns on nutritional value, integration into school meal and community nutrition programs, provision of subsidies or incentives for farmers, inclusion in national dietary guidelines, and endorsement through functional food labeling (FAO, IFAD, UNICEF, WFP and WHO, 2021; Fortune Business Insights, 2024; Coherent Market Insights, 2025). Collectively, these initiatives aim to bridge the gap between production and consumer demand, facilitating the wider acceptance of pseudocereals as mainstream dietary staples. **Figure 5** illustrates the SWOT analysis of pseudocereals in sustainable food systems.

## Conclusion

Globally, crops resilient to climate change stress factors are crucial, especially in resource-scarce and arid regions, to address challenges of food and nutritional security. Climate change is becoming challenging worldwide, particularly in arid regions where water and fertile land resources are limited. These climate change stress factors worsen the situation, threatening food security and sustainability. Currently, over 2 billion people around the world depend on staple crops such as maize, wheat, and rice. These crops do not completely fulfill the nutritional requirements, and this causes nutrient deficiencies referred to as “hidden hunger”. Moreover, the global population is expected to reach 10 billion by 2050, increasing the demand for food and nutrition. Only depending on staple crops is not enough to combat climate change and global population increase; there is a need to explore alternatives for these staple crops. Pseudocereals can be an alternative option, being nutrient-dense, packed with vitamins, minerals, and proteins, and with numerous health benefits. Currently, these crops share almost 15 bn market of the grain industry worldwide, which is expected to have CAGR of 7% in the next 8 years. Additionally, consumer demand has also increased because of increased awareness for nutrient-specific foods, usage and consciousness for ethically grown foods, need for plant-based proteins, and frequency of food delivery systems. This will point to a need to have more innovative and new products such as gluten-free items, pet food, beverages, and high-protein, high-fiber, and high-micronutrient food items made up of different pseudocereals. Major stakeholders in the super-grain industry have recently committed substantial investments toward expanding the portfolio of pseudocereal-based products, indicating the anticipated significance and growing demand for these crops in the near future (https://www.marketreportanalytics.com/reports/supergrains-260443#summary). These crops can withstand climate change stress factors, making them suitable for cultivation in arid regions. Research studies have shown that even small amounts of pseudocereals in our daily meals can enhance their nutritional value. Many varieties of pseudocereals have shown promising results in extreme conditions. Advanced molecular techniques such as high-throughput phenotyping, genome sequencing, nutritional profiling, gene editing, transcriptomics, marker-assisted breeding, and functional genomics will be pivotal in improving yield, quality, and stress tolerance. Researchers better understand the key characteristics in developing nutrient-rich and climate-resilient pseudocereals. Furthermore, initiatives to make pseudocereals familiar through awareness programs, policies to promote it, and sustainable farming practices can help in integrating pseudocereals into modern agriculture. Ultimately, pseudocereals are hidden treasures of arid regions, offering a sustainable, climate-resilient solution to food and nutritional insecurity. Their integration into modern agriculture could diversify food systems, reduce dependence on vulnerable staple crops, and empower smallholder farmers in resource-scarce areas. Incorporating pseudocereals into school meal programs, community nutrition initiatives, and local value chains could directly combat hidden hunger while fostering rural livelihoods. Future research should focus on region-specific breeding strategies, consumer acceptance, and sustainable agronomic practices to ensure large-scale adoption. By acting now—through coordinated policy, awareness, and innovation—pseudocereals can shift from being an underutilized resource to a cornerstone of global food security in a changing climate.
